# Cell Fate Control by Translation: mRNA Translation Initiation as a Therapeutic Target for Cancer Development and Stem Cell Fate Control

**DOI:** 10.3390/biom9110665

**Published:** 2019-10-29

**Authors:** Hyun-Jung Kim

**Affiliations:** Laboratory of Molecular Stem Cell Pharmacology, College of Pharmacy, Chung-Ang University, Seoul 156-756, Korea; hyunjungkim@cau.ac.kr; Tel.: +82-2-820-5619

**Keywords:** cancer, cell fate, differentiation, proliferation, stem cell, translation

## Abstract

Translation of mRNA is an important process that controls cell behavior and gene regulation because proteins are the functional molecules that determine cell types and function. Cancer develops as a result of genetic mutations, which lead to the production of abnormal proteins and the dysregulation of translation, which in turn, leads to aberrant protein synthesis. In addition, the machinery that is involved in protein synthesis plays critical roles in stem cell fate determination. In the current review, recent advances in the understanding of translational control, especially translational initiation in cancer development and stem cell fate control, are described. Therapeutic targets of mRNA translation such as eIF4E, 4EBP, and eIF2, for cancer treatment or stem cell fate regulation are reviewed. Upstream signaling pathways that regulate and affect translation initiation were introduced. It is important to regulate the expression of protein for normal cell behavior and development. mRNA translation initiation is a key step to regulate protein synthesis, therefore, identifying and targeting molecules that are critical for protein synthesis is necessary and beneficial to develop cancer therapeutics and stem cells fate regulation.

## 1. Introduction

A protein mediates the function of a gene. To produce proteins, genetic information stored in DNA has to be transcribed into mRNA. Then, the mRNA should be translated to generate proteins. However, the mRNA levels do not necessarily correlate with those of the protein [1,2,3,4]. There are multiple steps at which protein production is regulated, including transcriptional regulation, control of mRNA stability, translational regulation, and protein degradation [5,6]. Recent emerging data suggest that the regulation of protein synthesis is related to cancer development and that the control of translation efficiency is important for the determination of stem cell fate [1,5,7,8]. The current review focuses on the role of protein synthesis initiation on cell fate decision.

## 2. Translation Initiation and Regulation of Protein Synthesis

**Figure 1:**
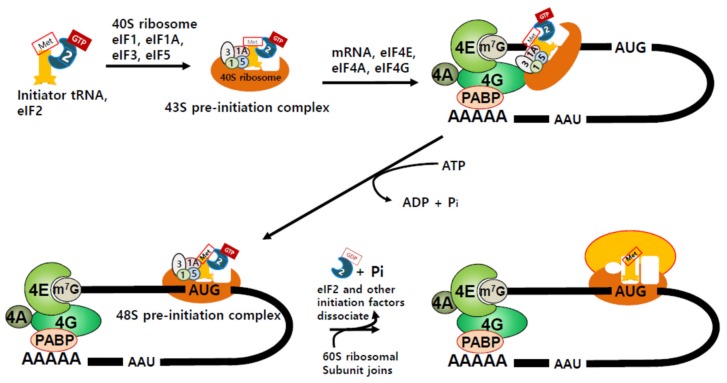
Cap-dependent translation initiation. When an initiator tRNA binds to guanosine triphosphate (GTP)-bound eukaryotic initiation factor (eIF2), the complex can further recruit eIF1, eIF1A, eIF3, eIF5, and 40S ribosome to form 43S pre-initiation complex (43S PIC). eIF4F, composed of eIF4A, eIF4E, and eIF4G, facilitates the recruitment of mRNA to 43S PIC. Then, eIF4E binds to the 5′-cap of mRNA, and eIF4G recruits poly(A)-binding protein (PABP), which binds to poly(A) on mRNA on the 3′-end, thus circularizing the mRNA to stabilize it for translation. eIF4A functions as a helicase and may facilitate mRNA scanning to find the initiation codon AUG. Once the codon is found by 43S PIC, eIFs are released and the 60S ribosomal subunit joins the assembly to generate protein synthesis-ready 80S ribosome ready for translation elongation.

Proteins are synthesized by the ribosome, which is composed of two subunits, a big subunit and a small subunit. In eukaryotes, these are the 60S (big) subunit and 40S (small) subunit [9]. Both big and small subunits comprise several RNA molecules (rRNAs) and many different proteins [10]. The two ribosomal subunits are separate when they are not involved in protein synthesis, but assemble near the 5′-end of mRNA to produce proteins [11]. Translation can be divided into four steps; initiation, elongation, termination, and ribosome recycling [11]. During initiation, protein synthesis-competent ribosomes are assembled and recognize the start codon. Protein synthesis is predominantly regulated at this step [12,13]. Aminoacyl transfer RNAs (aminoacyl-tRNAs) harbor an anticodon that deciphers the nucleotide sequence of mRNA, and bring the appropriate amino acids needed for protein synthesis to the ribosome [14]. Methionyl initiator tRNA (tRNAi Met) first binds to GTP-bound eukaryotic initiation factor 2 (eIF2), which then assembles with eIF1, eIF1A, eIF3, and eIF5, to form a 43S pre-initiation complex (43S PIC) [12] (Figure 1).

Interestingly, it has been suggested that protein synthesis is regulated differently in different mammalian somatic cells and is affected either globally or in a gene-specific manner [7,8]. Global translational control is mainly mediated by the modulation of factors that are involved in translation, whereas mRNA-specific control is associated with the code embedded in mRNA regulatory regions, such as the 5′- and/or 3′-untranslated regions (UTRs) (reviewed in [8,15,16]. Upstream open reading frames (uORFs) located in the 5′-regulatory region hinder protein synthesis of the main ORF (reviewed in [16]). In addition, secondary structure generated in the 5′-region by high GC content impedes translation by blocking the progression of the ribosomal complex [16,17]. Hence, depending on the 5′-region sequences of each mRNA, the efficiency of protein synthesis can be specifically regulated.

During mRNA transcription, after approximately 25–30 nucleotides are transcribed, the γ-phosphate of the 5′-triphosphate is removed from the transcript by RNA triphosphatase, and guanosine monophosphate (GMP) is transferred to the transcript from guanosine triphosphate (GTP), to form a cap [18]. Then, the N7 amine of the guanine cap is methylated to yield m7G-capped 5′ mRNA (reviewed in [18]). For cap-dependent translation, this structure recruits the eIF4F complex, which contains the cap-binding protein eIF4E, DEAD-box helicase eIF4A, and a scaffold protein eIF4G that facilitates the attachment to m7G-capped 5′-end of mRNA [18] (Figure 1). Then, the aforementioned 43S PIC is recruited to mRNA via the eIF4F complex and scans through the 5′ UTR of mRNA until it recognizes the start codon AUG (Figure 1). Once AUG is recognized, eIF2 and other initiation factors detach from the mRNA, and the 60S big ribosomal subunit is recruited [11,16]. The completed protein synthesis-competent ribosome then starts to synthesize the protein [11,12,16] (Figure 1). The initiation regulates the rate of translation and, thus, is considered as the main step for translational control [12,13]. Therefore, initiation factors involved in this process are considered to be important for the control of protein synthesis, and mutations in the genes or the altered expression of these factors are associated with cancer development, progression, and cell fate determination.

## 3. Molecular Signals that Regulate Protein Synthesis

### 3.1. Mitogen-Activated Protein Kinase Kinase (MEK) Signals and Protein Synthesis

**Figure 2:**
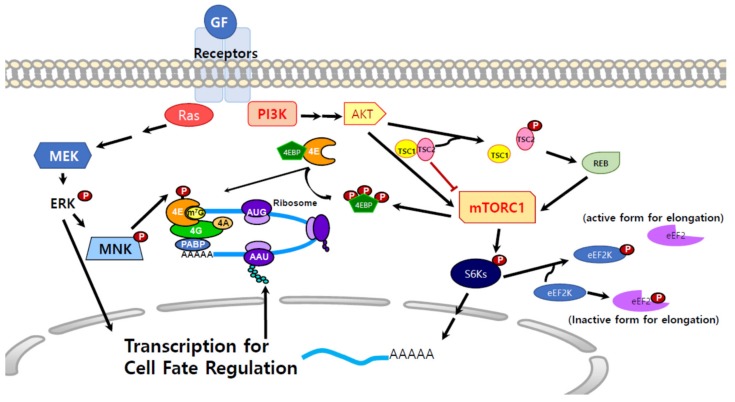
Signaling pathways that influence protein synthesis. Signals that are activated by growth factors not only affect transcription but also translation. Mitogen-Activated Protein Kinase Interacting Protein Kinases (MNKs) can be phosphorylated by activated extracellular signal-regulated kinases (ERKs) and further phosphorylate eIF4E, which is important for recruiting mRNA for translation initiation. Binding of the growth factors to the receptors activates phosphatidylinositol 3-kinase (PI3K) and AKT. Activated AKT phosphorylates tuberous sclerosis complex 2 (TSC2), which leads to the dissociation of the TSC1/ TSC2 complex that negatively regulates mammalian target of rapamycin complex 1 (mTORC1) and leads to mTORC1 activation. mTORC1 hyper-phosphorylates 4EBP, which results in the release of eIF4E for protein synthesis. Activated mTORC1 also activates S6Ks, which further activate eukaryotic translation elongation factor 2 (eEF2) and facilitate elongation.

Protein synthesis is necessary for the biology of the cell, including growth, survival, proliferation, and differentiation, and is the final process in the generation of the functional molecules, proteins. Therefore, various signal pathways converge at and regulate translation [1,19,20,21] (Figure 2). One of the signaling pathways that affect the translational machinery is the typical growth or proliferation signal mediated by MEKs [1,22]. MEK activates extracellular signal-regulated kinases (ERK) and p38 mitogen activated protein kinases (MAPK), which then phosphorylate MAPK-interacting kinases (MNKs) MNK1/MNK2 (Figure 2) [23,24]. MNKs phosphorylate eIF4E (Figure 2) [1,23,25]. It has been suggested that MNK1 is recruited to eIF4F by binding to eIF4G and phosphorylates Ser209 of eIF4E [26,27]. Although it is still being debated, eIF4E phosphorylation is likely correlated with protein synthesis [1,22,28]. The phosphorylation level of eIF4E diminishes upon heat shock, a condition that represses protein synthesis, and hypo-phosphorylation of eIF4E appears to be proportional to the reduction of translation [29]. Recently, it has been shown that eIF4E phosphorylation by MNKs in the presence of EGF and FGF2 in neural progenitor cells (NPCs) increases lysine demethylase 5A (KDM5A) protein synthesis; conversely, in the presence of ciliary neurotrophic factor (CNTF), Signal transducer and activator of transcription 3 (STAT3) is activated instead of MEK, eIF4E is no longer phosphorylated, and KDM5A protein synthesis is inhibited even when the *Kdm5a* mRNA levels increase, suggesting a positive correlation of eIF4E phosphorylation with protein synthesis [1]. Crystal structure analysis and biophysical studies revealed that the binding affinity of phosphorylated eIF4E to the mRNA cap is lower than that of unphosphorylated eIF4E, and it has been proposed that phosphorylation might play an important role in releasing eIF4E from the eIF4F complex and recycling eIF4E for the next round of translation [30,31].

### 3.2. mTOR Signals and Protein Synthesis

Another important pathway that regulates protein synthesis is the mammalian target of rapamycin (mTOR) pathway [32,33]. mTOR is a Ser/Thr kinase that mediates signal transduction downstream of the phosphatidylinositol 3-kinase (PI3K)/AKT or Ras/MAPK pathways. It links extracellular signals with intracellular energy status and controls protein synthesis to modulate cell proliferation and growth [33,34] (Figure 2). Once AKT is activated by upstream signaling, it phosphorylates and disrupts the tuberous sclerosis complex 2 (TSC2)/TSC1 heterodimer, which functions as a negative regulator of mTOR [35,36,37]. TSC2 is phosphorylated by AKT and the phosphorylated form can no longer interact with TSC1, which leads to the activation of S6K and phosphorylation of eukaryotic translation initiation factor 4E-binding protein (4EBP) [37]. TSC2 not only binds to TSC1 but also functions as a GTPase-activating protein (GAP) [38]. Dissociation of TSC1 and TSC2 activates Ras homolog enriched in brain (RHEB), a small GTPase that functions upstream of mTOR in response to PI3K/Akt (Figure 2) [39]. The main downstream targets of mTOR are the translational machinery and ribosomal components [33]. mTOR binds to various types of partner proteins, forming two complexes: mTOR complex 1 (mTORC1) and mTORC2 [34]. mTORC2 is composed of mLST8, the protein rapamycin-insensitive companion of mTOR (Rictor), Protor, and mammalian stress-activated protein kinase (SAPK) interacting protein [mSIN1] [33,34,40]. mTORC2 controls cell survival and cytoskeletal reorganization [40]. As mRNA translation is a high-energy consuming process, the cell precisely regulates it through mTORC1 by sensing the growth factor signals and nutrients levels [34]. Thus, mTORC1 controls cell biology, including cell proliferation, differentiation, survival, growth, protein synthesis, and autophagy [33,41]. mTOR, mLST8, the DEP domain-containing mTOR-interacting protein (DEPTOR), a 40-kDa Pro-rich AKT substrate (PRAS40), TTI1/TEL2, and the regulatory associated protein of mTOR (Raptor) are known components of mTORC1 [42,43,44]. Raptor brings 4EBP, S6K1/2 and other molecules to mTORC1. Thereupon, mTOR phosphorylates its substrates, which results in the activation of protein synthesis [44,45].

eIF4E can bind the unphosphorylated 4EBP which has homologous sequence of eIF4G; 4EBP suppresses translation by competing with eIF4G for eIF4E and hinders eIF4F complex formation [46,47,48]. For the translation initiation to take place, 4EBP needs to be dissociated from eIF4E, which can be triggered by extracellular stimuli, such as growth factors and hormones, and leads to the phosphorylation of 4EBP through PI3K and the mTOR pathway [33]. Hyper-phosphorylated 4EBP dissociates from eIF4E and increases eIF4E availability for the formation of the eIF4F complex for translation [49,50]. When S6Ks are activated by mTORC1, they promote the translation of mRNAs that encode the necessary components of the translational machinery, including ribosomal proteins, elongation factors (EFs), and poly(A)-binding protein [33]. S6Ks also phosphorylate and inhibit eukaryotic translation elongation factor 2 kinase (eEF2K) whose role is to phosphorylate and inhibit eEF2 [51]. Hence, external stimuli by growth factors or hormones, result in S6K activation, and further induce the phosphorylation and inhibition of eEF2K, which increase eEF2 activity and elongation rates during protein synthesis [52,53,54]. Faced with various stimuli, the cell needs to modulate the translational machinery, and adjust the rate and amount of protein production to adapt to the changing environment, suggesting that appropriate regulation of translation is critical for cell biology and behavior.

## 4. Protein Synthesis and Cancer

### 4.1. eIF4E and Cancer

Dysregulation of protein synthesis is highly correlated with cancer development [11,28,41,55,56]. High protein expression appears to be responsible for the resistance to cell death and increased cell proliferation [28,57,58]. One of the candidate signaling molecules that is involved in cancer progression is eIF4E, the cap-binding component of eIF4F, at which the growth and proliferation signals converge, making it an important factor that controls cell normality [50,59,60]. Interestingly, eIF4E levels are relatively low in HeLa cells, approximately 0.8 × 106 molecules per cell [29]. By contrast, another component of the eIF4F complex, eIF4A is present more than 10-fold molar excess [15,29,61]. The factor to ribosome ratio is 0.8 for eIF2, 0.6 for eIF3, and 3.0 for eIF4A [62]. The relative limitation of eIF4E levels in the cell renders it an important factor for translation control, and its aberrant expression is associated with cancer development [11,41]. Notably, eIF4E is regulated by MYC, based on the presence of MYC-binding motifs in the promoter of eIF4E gene and the mutation of these motifs inactivates the eIF4E promoter [63,64]. In addition, dominant negative c-MYC mutant suppresses transcription of the eIF4E gene [63,64]. Interestingly, c-MYC and eIF4E appear to regulate each other, such that upregulation of eIF4E expression increases the translation of c-MYC [11,65]. In MYC-transformed B cells, eIF4E is upregulated and activates the translation of phosphoribosyl-pyrophosphate synthetase 2 (PRPS2), which results in the promotion of nucleotide biosynthesis [66]. In the 5′ UTR of PRPS2, the pyrimidine-rich translational element that is known to be regulated by eIF4E is present and indeed the translation of PRPS2 is controlled by eIF4E [66,67]. PRPS2 produces nucleotide biosynthetic precursor, 5-phosphoribosyl-1-pyrophosphate, therefore, it is beneficial for cancer cell proliferation and survival if the expression of PRPS2 is elevated [66].

As mentioned above, eIF4E phosphorylation is regulated by MNKs that act downstream of the MEK/ERK and p38 MAPK [68]. Mnks generally function downstream of MAPKs but, according to a recent report, MNK2a activation leads to activation of p38 MAPK, which serves as a tumor suppressive signal [69]. Serine 209 of eIF4E is phosphorylated by MNKs, and the phosphorylation stimulates translation of a subset of mRNAs that play roles in cell proliferation and survival. These mRNAs possess specific regulatory features, such as lengthy and GC-rich structured 5′ UTRs [16,20,28]. The genes regulated by hyper-phosphorylated eIF4E enhance malignancy, e.g., CYCLIN D1, VEGF, MMP-3, SURVIVIN, c-MYC and FGF2 [60,70,71,72,73]. Notably, aberrant expression of a set of mRNAs that are involved in cell proliferation, survival, or promotion of cancer is highly affected by eIF4E-mediated translation [60,71,72]. The overexpression of eIF4E causes tumorigenic transformation in various types of cells [59,74,75,76]. Co-culture of multiple myeloma (MM) cell lines with mesenchymal stem cells (MSCs) derived from MM patients revealed that, in contrast with co-culture with MSCs from healthy donors, protein synthesis, especially eIF4E/eIF4GI and cell proliferation, is upregulated by co-culture with MSCs from MM patients [77].

Similar to the regulation of translation by eIF4E levels, eIF4E phosphorylation regulates protein synthesis; however, it enhances the synthesis of a subset of proteins, without affecting global protein synthesis [1,20,41,73]. MCL, MMP3, SNAIL, and the recently identified KDM5A are examples of such regulated proteins [1,73,78,79]. Interestingly, eIF4E also plays a non-translational role, in transporting some mRNAs from the nucleus to the cytoplasm [80]. In various cancer cells originating from the breast, colon, prostate, lung, skin, and bladder, enhanced eIF4E is commonly observed [75,81,82,83,84]. In addition, increased phosphorylation of eIF4E is responsible for certain tumorigenic genes and correlates with progression of prostate cancer [28]. Hence, recently, eIF4E has been considered as the main target for the development of cancer therapy [28,81,85]. Numerous experiments were performed to identify small molecules that downregulate transcription of the eIF4E gene, reduce the stability the eIF4E transcript, interrupt binding of eIF4E to its binding partners, and block its binding to cap-mRNAs (reviewed in [85]). For example, since the availability of eIF4E is regulated by 4EBP, agents that function similarly to 4EBP have been developed to prevent cap-dependent translation [86].

However, surprisingly, it has been suggested that normal levels of eIF4E is responsible for cancer transformation [59]. A recent study of an *Eif4e*+/− mouse revealed that reduced expression of eIF4E is compatible with normal development [59]. Interestingly, in the same study, the eIF4E dose played an essential role in cellular transformation [59]. When introduced with *Ras* and *Myc*, *Eif4e*+/− mouse embryonic fibroblasts (MEFs) became resistant to tumor transformation, and the global protein synthesis rate was not altered compared to wild-type MEFs treated in the same manner [59]. It appears that a subset of genes (133 genes) that mediate the function of proteasome, cellular transduction, generation of reactive oxygen species, and nucleotide biosynthesis are affected by reduced eIF4E levels during oncogenic transformation [59]. Furthermore, genes that regulate the production or levels of reactive oxygen species are associated with cell transformation and the expression levels of these genes are reduced in haploinsufficient *Eif4e*, resulting in resistance to transformation [59]. Interestingly, 5′ UTRs of these genes do not possess features predisposing to eIF4E-mediated translation, e.g., length, high GC content, and complex secondary structure. Instead, they have a cytosine-rich 15-nucleotide motif in the 5′ UTR, termed the cytosine-enriched regulator of translation (CERT) domain [59]. Intriguingly, normal levels of eIF4E actually aid the cellular transformation. Reduction of eIF4E levels by using shRNA reduced soft agar colony formation of human cancer cells [59]. Although it is unclear why cells produce more eIF4E than is needed, it appears that cancer cells use this to benefit their survival. High expression of eIF4E appears to play an important role in breast cancer development and is correlated with breast cancer metastases [56,81,87]. For instance, ribavirin, an antiviral guanosine analogue that competes with 5′-cap and inhibits eIF4E activity, inhibits the proliferation and clonogenic potential of breast cancer cell lines with elevated eIF4E levels [81,88].

### 4.2. mTOR, 4EBP, and Cancer

mTOR is a downstream molecule that mediates the PI3K and AKT signals. It has been reported that thymocytes derived from transgenic mouse with constitutively activated AKT2 have increased cell size and protein synthesis [58]. Phosphorylation of 4EBP is required for such increase and the resultant hyperactivation of eIF4E is essential for T-cell lymphomagenesis, partially increasing the expression of anti-apoptotic protein Mcl-1 [58]. PP242, an ATP active-site inhibitor of mTOR, inhibits eIF4E hyperactivation and could be a therapeutic drug for treating lymphoma [58]. It has been suggested that inhibiting mTOR signaling could suppress cancer invasion and metastasis [67]. Hsieh and colleagues demonstrated that mTOR enhances the migration and invasion of prostate cancer cells by affecting certain genes that are controlled by translation, and developed an ATP site inhibitor of mTOR that prevents prostate cancer metastasis [67]. mTOR ATP site inhibitor targets mTOR-dependent 4EBP1 phosphorylation, thereby recruiting eIF4E to hypo-phosphorylated 4EBP1 and preventing the formation of eIF4F complex [67]. Recent advances in the development of inhibitors that affect translation have been reviewed, and the effects on cancer cells and metastasis have been thoroughly summarized in a paper by Bhat et al. [11].

### 4.3. eIF2 and Cancer

eIF2, a GTPase, is composed of α, β, and γ subunits, and also involved in the regulation of translation [89]. Particularly, the α subunit is phosphorylated at serine 51, which controls translation in cells under stressful conditions and is involved in cancer development [89,90,91]. In tumor cells that are exposed to various stimuli, including hypoxia, lack of nutrients, and those induce DNA damage, endoplasmic reticulum (ER) stress and unfolded protein response are produced [92]. The ER stress induces downstream eIF2α phosphorylation on serine 51 via kinases such as PERK, protein kinase RNA-activated (PKR), general control non-derepressible 2 (GCN), and heme-regulated inhibitor (HRI) [92,93,94]. As mentioned above, GTP-bound eIF2 plays an important role in translation initiation by forming a ternary complex with tRNAi Met and mRNA. During mRNA scanning, GTP is hydrolyzed to GDP and Pi upon recognition of the start codon, and GDP-bound eIF2 is released from the complex. For another round of protein synthesis to occur, eIF2B functions as a guanine exchange factor, and replaces GDP with GTP on eIF2 [95]. However, the phospho-eIF2α is unable to form a ternary complex for protein synthesis therefore, global translation is attenuated [96,97]. Phosphorylated eIF2α also inhibits eIF2B, which results in the inhibition of global protein synthesis, while increasing translation of a subset of mRNAs [89,95]. Furthermore, mRNAs that are positively regulated by the phosphorylated eIF2α possess uORFs in 5′ UTR, which hinder the identification of ORFs [92]. It has been suggested that eIF2α phosphorylation somehow overcomes the hindrance of uORFs and facilitates the translation of certain genes, e.g., ATF4, CHOP, and GADD34, leading to cell survival and growth [98,99]. In addition, eIF2α phosphorylation increases the translation of genes that have internal ribosome entry site (IRES) in the 5′ UTR [100], which will be addressed in Section 4.4.

In the tumor tissue of female breast cancer patients, phospho-eIF2α is substantially upregulated when compared with that in the peritumor tissues [101]. Similarly, increased levels of eIF2α have been detected in Hodgkin lymphoma, bronchioloalveolar cancer cells, thyroid cancer cells, gastrointestinal carcinomas, benign and malignant melanocytoma, and colon carcinoma [102,103,104,105,106]. The role of phopho-eIF2α in cancer cell is still being debated. High expression and phosphorylation of eIF2α may lead to increased levels of a cohort of proteins that are important for cell survival and proliferation, and may result in tumor growth [98,99]. However, it has been suggested that depending on the duration of the ER stress, the survival signal can be converted to facilitate cell death [99]. Interestingly, it has been recently suggested that phospho-eIF2α plays an important role in the inhibition of triple-negative breast cancer growth [101]. Reduced tumor infiltration of the lymph nodes is observed in patients with elevated phospho-eIF2α levels, suggesting that eIF2α indeed plays a dual role in cellular survival, and partner molecules and detailed mechanisms that culminate in such differential effects should be identified for cancer treatment [101].

### 4.4. Cap-Independent Translation and Cancer

Recent emerging data suggest that in addition to factors that regulate the assembly of or recruit the translation-competent ribosome to the 5′-cap of mRNA, ribosomal components are also directly linked to cancer development [107]. Although the majority of protein synthesis initiation depends on mRNA recognition by eIF4E that is 5′-cap–dependent, some proteins can be produced bypassing this step. A specific IRES sequence in the 5′ UTR directly recruits the 40S ribosome to mRNA [108,109,110]. A unique RNA structure known as a cap-independent translational enhancer (CITE) also contributes to the initiation of protein synthesis that is independent of cap and IRES [108,111]. Furthermore, N6-methyladenosine residues in the 5′ UTR recruit eIF3 and the 40S ribosome, and initiate cap-independent translation [108].

It has been suggested that translation of several proteins, including growth factors, survival- and/or death-related proteins, and oncogenes, relies on cap-independent IRES elements under certain circumstances [108,112,113,114,115,116]. Historically, IRES was first discovered in the 5′ UTR of poliovirus mRNAs that were effectively translated even without eIF4E binding [109]. Interestingly, in eukaryotic genomes, including the human genome, several genes appear to be translated using cap-independent sequences [110,117]. It has been suggested that 5–10% of mRNAs are translated by cap-independent translation [117,118]. Recently, an unbiased screening of human mRNAs using a high-throughput bicistronic assay revealed an enrichment of regulatory elements for cap-independent translation not only in the 5′ UTRs but also in the 3′ UTRs [117]. This implies that the ribosome can be recruited to the 3′ UTR, enhancing protein synthesis from the upstream sequence [117]. Intriguingly, mRNAs that contain IRES are preferentially translated when cap-dependent translation is inhibited, during cell differentiation, proliferation, and under hypoxic conditions and nutrient limitation [119]. A recent PubMed search and clustering analysis revealed that approximately 21% of transcription factor mRNAs, 15% of growth factor mRNAs, and 22% of receptor and transporter mRNAs are prone to be translated via an IRES-dependent way [108]. Examples of such mRNAs are c-MYC, HIF1a, FGF, VEGF, and CAT-1 [65,120,121,122,123]. However, more evidence is needed to propose a general mechanism or concept for the cellular role of IRES because of some controversial data and views on the subject [124].

CITEs have been identified in the 5′ UTR or 3′ UTR of mRNAs, and serve to recruit some initiation factors [108,111,124,125]. Different from IRES-dependent translation, CITEs-mediated protein synthesis, CITEs recognize and bind essential initiation factors to begin translation [108,124]. It has been reported that, upon etoposide treatment to induce apoptosis, *Apaf-1* mRNA translation proceeds efficiently, although the 5′-cap is absent, via CITE-dependent translation [126]. No IRES-dependent translation appears to proceed under such conditions [126].

## 5. Translational Control of the Stem Cell Fate

### 5.1. Differences in the Rate and Amount of Translation in Stem and Differentiated Cells

The observations that aberration of protein synthesis is linked to the development of cancer raise a question of whether the typical role of mRNA translation is cell fate determination. Not much stem cell research has been done from the point of view of translation; however, recent data show that protein synthesis rates and machinery are different in stem cells, progenitor cells (PCs), and differentiated somatic cells [1,7,127,128]. During development, appropriate numbers and types of cells should be generated, and the process should be precisely orchestrated with changes in the local environment, in a time- and location-dependent manner. Transcriptional regulation has been extensively studied to identify the mechanisms that underlie control of the stem cell fate [129,130]. However, it is difficult to resolve some transcript and protein level mismatches observed during the development or differentiation of stem cells or PCs by transcriptional regulation only [1,131]. Increasing evidence suggests that translation is an essential step controlling the stem cell fate [1,7,127,132]. In the current section, exciting recent studies that revealed translational differences in controlling features of stem cells and PCs are highlighted. In addition, recent articles that unravel the underlying molecular mechanisms are introduced.

As reported by Ingolia et al., who undertook deep-sequencing of the ribosome-protected mRNA fragments in mouse embryonic stem cells (ESCs), the mammalian proteome is more complex than initially assumed [133]. By using the drug harringtonine, which causes ribosomes to accumulate at translation sites, and analyzing the translation data, Ingolia et al. showed that a wide range of unannotated or modified ORFs exist [133]. In addition, mouse ESCs produced atypical protein-coding transcripts [133]. Another recent study revealed that hematopoietic stem cells (HSCs) synthesize less protein than hematopoietic progenitor cells, suggesting the existence of distinct translation control mechanisms in specific cell types [127]. Altered protein synthesis of HSCs derived from a ribosomal protein loss-of-function mouse, *Rpl24Bst*/+, in which translation rate is reduced to approximately 30% that in the wild-type mouse, resulted in reduced cell proliferation and reconstitution of differentiated hematopoietic cells after transplantation into irradiated mouse [127,134]. Furthermore, a conditional deletion of Pten in HSCs increased protein synthesis, which was blocked by the introduction of *Rpl24* mutation, and resulted in the restoration of HSC multi-potency [127]. This clearly suggests the involvement of translation in HSC fate control. Interestingly, phosphorylation of 4EBP, the negative regulator of eIF4E, is lower in HSCs than in other hematopoietic cells [127,134]. Similarly, the translation rate and amount of protein synthesis in mouse epidermal SCs are lower than those in the immediate PCs in vivo, as determined by measuring the incorporation of O-propargylpuromycin into actively synthesized proteins [135]. In the mouse epidermal SCs, differentiation but not proliferation is correlated with increased protein synthesis [135]. Interestingly, RNA methylation appears to play a role in the control of translation. NSUN2, an RNA methyltransferase, protects tRNA from cleavage, and this induces protein synthesis and cell commitment to differentiate in epithelial SCs from K5-SOS mouse, an animal model for human squamous tumors [135]. Another recent report demonstrated that regulation of ribosome biogenesis and protein synthesis is important for the control of germline stem cell (GSC) differentiation [136]. In the report, authors performed an unbiased *in vivo* transcriptome-wide 8,171 RNAi screening in female *Drosophila* GSCs and found that transition from self-renewal to differentiation relies on enhanced ribosome biogenesis and increased protein synthesis [136].

### 5.2. eIF4E, 4EBP, and the Control of Stem Cell Fate

It appears that a set of genes is regulated by translation factors. Modulation of these factors affects the translation of targets, leading to the synthesis of a unique pool of proteins, and determines the identity of a cell. As mentioned above, eIF4E is a component of the eIF4F complex. Until the discovery of variants in wheat germ cells, it has been assumed that eIF4E is the only isoform of the protein [137]. The existence of isoforms in other species has been also reported, and the eIF4E family is now divided into three classes depending on the core sequence homology and the binding partners (reviewed in [138]). It is well known that eIF4E1 binds to the m7G-cap mRNA. Interestingly, eIF4E2 shows weak binding to the cap, and is suggested to mediate translation under hypoxic conditions in tumors [57,139]. Furthermore, eIF4E3 lacks essential amino acids that are required for m7G-cap binding; however, it was recently reported to bind to the cap in a distinct manner and inhibit tumor progression [140]. The availability of eIF4E appears to be important for the regulation of translation in SCs. A recent study revealed the occurrence of eIF4E1 and 4E-T in granules with the processing body proteins Lsm1 and Rck in neural precursors [141]. It appears that eIF4E1 is sequestered in P-body–like granules in neural precursors by its binding partner 4E-T, which represses the translation of proteins essential for neurogenesis [141]. The complex targets mRNAs that encode transcription factors and differentiation-related proteins, including proneurogenic bHLH mRNAs, and have been translocated to the granules, thus inhibiting further translation [141]. Therefore, disruption of the complex leads to the generation of premature neurons and neural precursor depletion [141].

Not only global translation but also gene-specific translation is important for stem cell or PC fate determination. In the neural progenitor/neural stem cells (NSCs), it has been recently shown that KDM5A, a histone demethylase, is involved in the maintenance of multipotency by repressing the differentiation of NPCs or NSCs into astrocytes [1]. Intriguingly, mRNA levels of *Kdm5a* in NSCs/NPCs are lower than those in differentiated astrocytes, with opposite trends shown by protein levels [1]. NSCs proliferate in the presence of EGF and FGF2, which triggers the activation of ERK and MNK, and phosphorylation of eIF4E [1]. However, in the absence of those mitogens and in the presence of CNTF, NSCs/NPCs differentiate into astrocytes by activating the JAK and STAT3 signaling and shutting off ERK and downstream signaling, thus halting eIF4E phosphorylation [1]. Therefore, regardless of the high levels of *Kdm5a* transcripts, translation no longer occurs. Next, because of insufficient KDM5A protein levels, H3K4 demethylation at *Gfap* promoter does not proceed, therefore GFAP expression increases to convert NSCs to become astrocytes [1].

One of the mechanisms that control protein synthesis in HSCs is the regulation of 4EBP phosphorylation. Consistent with the data for eIF4E, in double knockout mouse (DKO) lacking *4Ebp1* and *4Ebp2*, translation of a subset of genes is reduced, but global protein synthesis is not affected, probably because of an increased availability of free eIF4E for eIF4F complex formation [132]. It has been reported that adult HSCs harbor more hypo-phosphorylated 4EBP and 4EBP2 molecules than most other hematopoietic progenitors [128]. Hypo-phosphorylated 4EBP would bind eIF4E and prevent the formation of the eIF4F complex, which may result in the reduced synthesis of a subset of proteins in HSCs. Although Yin-yang2 (YY2) is essential for mouse (m) ESC self-renewal, increased YY2 levels induce the differentiation of mESCs into cardiovascular lineages [132]. YY2 protein levels increase up to 2.4-fold in in *4Ebp1 4Ebp2* DKO mouse, with no difference in *Yy2* mRNA levels between the DKO and wild-type mice, suggesting that translation efficiency of YY2 negatively correlates with 4EBP expression [132]. Interestingly, four splicing variants of *Yy2* have been identified, two of which have an intron in the 5′ UTR [132]. The retained intron appears to provide a lengthy, GC-rich, and structured 5′ UTR, and is, therefore, prone to regulation by eIF4E. Hence, it is plausible to speculate that in *4Ebp1 4Ebp2* DKO mouse, the efficiency of translation of the *Yy2* splicing variants that contain the intron increases [132].

An important role of the cap-independent translation in stem cell fate determination has been reported. In human (h) ESCs, death-associated protein 5 (DAP5), a protein related to eIF4G but lacking the N-terminal portion and therefore unable to bind to either eIF4E or the poly(A)-binding protein, also determines the cell fate [142,143,144]. DAP5 recruits the ribosome directly to an mRNA that harbors IRES in the 5′ UTR and mediates translation of various proteins that are critical for cell differentiation [142]. It has been shown that *Dap5* knockdown results in defective differentiation and in persistent pluripotent gene expression during hESC differentiation [142]. Genes that are affected by DAP5-mediated translation are related to mitochondrial oxidative respiration and cell differentiation, including the chromatin modifier HMGN3, and knock-down of *Dap5* impairs ESC differentiation [142,145]. Interestingly, *Dap5* knockout mESCs do not differentiate and the ERK pathway is repressed in such cells [145]. The evidence that *Dap5* knockout mESCs showed impaired differentiation upon retinoic acid treatment, suggests a critical role of DAP5 and mRNA translation in differentiation [146]. The synthesis of the mitogen-activated protein kinase kinase kinase 3 (Map3k3) and son of sevenless homolog 1 (Sos1) is reduced in *Dap5* null mESCs [145].

A transcriptome-wide *in vivo* RNAi screening of the *Drosophila* germline revealed that ribosome biogenesis and protein synthesis are important for the transition between GSC self-renewal and differentiation [136]. Sanchez et al. identified 646 genes out of 8171 gene knockdowns that are required for germline development. High levels of rRNA transcription are observed in GSCs and, interestingly, reduction of rRNA levels alters cell morphology and inhibits production of MAD, a signal component of the bone morphogenetic protein pathway [136].

The detailed roles and effects of translation in stem cell biology have not yet been fully identified. Additional studies should provide insights into how transcription signals and signal transduction pathways converge in translation, and regulate stem cell proliferation, differentiation, and function.

## 6. Conclusions

Recent data have challenged the traditional view that transcription and translation are highly correlated. Translation of some genes that are highly transcribed is low and vice versa. These findings suggest that translation controls and regulates the proteome under certain biological conditions. Aberrant synthesis of proteins can transform cells to become malignant. In addition, during normal development, when stem cells or PCs differentiate into progeny, the rate and amount of protein synthesis are altered, and determine the cell fate. Understanding how cell fate is regulated at the translational level will provide insights for preventing or treating cancer as well as inducing stem cells to generate desired cell types.

## References

[1] Kong S.Y., Kim W., Lee H.R., Kim H.J. (2018). The histone demethylase KDM5A is required for the repression of astrocytogenesis and regulated by the translational machinery in neural progenitor cells. FASEB J..

[2] Scott P.A., Smith K., Poulsom R., De Benedetti A., Bicknell R., Harris A.L. (1998). Differential expression of vascular endothelial growth factor mRNA vs protein isoform expression in human breast cancer and relationship to eIF-4E. Br. J. Cancer.

[3] Rosenwald I.B., Kaspar R., Rousseau D., Gehrke L., Leboulch P., Chen J.J., Schmidt E.V., Sonenberg N., London I.M. (1995). Eukaryotic translation initiation factor 4E regulates expression of cyclin D1 at transcriptional and post-transcriptional levels. J. Biol. Chem..

[4] Saito H., Hayday A.C., Wiman K., Hayward W.S., Tonegawa S. (1983). Activation of the c-myc gene by translocation: A model for translational control. Proc. Natl. Acad. Sci. USA.

[5] Roux P.P., Topisirovic I. (2018). Signaling Pathways Involved in the Regulation of mRNA Translation. Mol. Cell. Biol..

[6] Radhakrishnan A., Green R. (2016). Connections Underlying Translation and mRNA Stability. J. Mol. Biol..

[7] Buszczak M., Signer R.A., Morrison S.J. (2014). Cellular differences in protein synthesis regulate tissue homeostasis. Cell.

[8] Truitt M.L., Ruggero D. (2016). New frontiers in translational control of the cancer genome. Nat. Rev. Cancer.

[9] Wilson D.N., Doudna Cate J.H. (2012). The structure and function of the eukaryotic ribosome. Cold Spring Harb. Perspect. Biol..

[10] Attardi G., Amaldi F. (1970). Structure and synthesis of ribosomal RNA. Annu. Rev. Biochem..

[11] Bhat M., Robichaud N., Hulea L., Sonenberg N., Pelletier J., Topisirovic I. (2015). Targeting the translation machinery in cancer. Nat. Rev. Drug Discov..

[12] Sonenberg N., Hinnebusch A.G. (2009). Regulation of translation initiation in eukaryotes: Mechanisms and biological targets. Cell.

[13] Richter J.D., Sonenberg N. (2005). Regulation of cap-dependent translation by eIF4E inhibitory proteins. Nature.

[14] Agris P.F. (2004). Decoding the genome: A modified view. Nucleic Acids Res..

[15] Duncan R., Etchison D., Hershey J.W. (1983). Protein synthesis eukaryotic initiation factors 4A and 4B are not altered by poliovirus infection of HeLa cells. J. Biol. Chem..

[16] Hinnebusch A.G., Ivanov I.P., Sonenberg N. (2016). Translational control by 5′-untranslated regions of eukaryotic mRNAs. Science.

[17] Svitkin Y.V., Pause A., Haghighat A., Pyronnet S., Witherell G., Belsham G.J., Sonenberg N. (2001). The requirement for eukaryotic initiation factor 4A (elF4A) in translation is in direct proportion to the degree of mRNA 5′ secondary structure. RNA.

[18] Ramanathan A., Robb G.B., Chan S.H. (2016). mRNA capping: Biological functions and applications. Nucleic Acids Res..

[19] Robichaud N., Sonenberg N. (2017). Translational control and the cancer cell response to stress. Curr. Opin. Cell Biol..

[20] Proud C.G. (2015). Mnks, eIF4E phosphorylation and cancer. Biochim. Biophys. Acta.

[21] Morita M., Gravel S.P., Hulea L., Larsson O., Pollak M., St-Pierre J., Topisirovic I. (2015). mTOR coordinates protein synthesis, mitochondrial activity and proliferation. Cell Cycle.

[22] Genheden M., Kenney J.W., Johnston H.E., Manousopoulou A., Garbis S.D., Proud C.G. (2015). BDNF stimulation of protein synthesis in cortical neurons requires the MAP kinase-interacting kinase MNK1. J. Neurosci..

[23] Waskiewicz A.J., Flynn A., Proud C.G., Cooper J.A. (1997). Mitogen-activated protein kinases activate the serine/threonine kinases Mnk1 and Mnk2. EMBO J..

[24] Scheper G.C., Morrice N.A., Kleijn M., Proud C.G. (2001). The mitogen-activated protein kinase signal-integrating kinase Mnk2 is a eukaryotic initiation factor 4E kinase with high levels of basal activity in mammalian cells. Mol. Cell. Biol..

[25] Fukunaga R., Hunter T. (1997). MNK1, a new MAP kinase-activated protein kinase, isolated by a novel expression screening method for identifying protein kinase substrates. EMBO J..

[26] Pyronnet S., Imataka H., Gingras A.C., Fukunaga R., Hunter T., Sonenberg N. (1999). Human eukaryotic translation initiation factor 4G (eIF4G) recruits mnk1 to phosphorylate eIF4E. EMBO J..

[27] Waskiewicz A.J., Johnson J.C., Penn B., Mahalingam M., Kimball S.R., Cooper J.A. (1999). Phosphorylation of the cap-binding protein eukaryotic translation initiation factor 4E by protein kinase Mnk1 in vivo. Mol. Cell. Biol..

[28] Furic L., Rong L., Larsson O., Koumakpayi I.H., Yoshida K., Brueschke A., Petroulakis E., Robichaud N., Pollak M., Gaboury L.A. (2010). eIF4E phosphorylation promotes tumorigenesis and is associated with prostate cancer progression. Proc. Natl. Acad. Sci. USA.

[29] Duncan R., Milburn S.C., Hershey J.W. (1987). Regulated phosphorylation and low abundance of HeLa cell initiation factor eIF-4F suggest a role in translational control. Heat shock effects on eIF-4F. J. Biol. Chem..

[30] Marcotrigiano J., Gingras A.C., Sonenberg N., Burley S.K. (1997). X-ray studies of the messenger RNA 5′ cap-binding protein (eIF4E) bound to 7-methyl-GDP. Nucleic Acids Symp. Ser..

[31] Scheper G.C., Proud C.G. (2002). Does phosphorylation of the cap-binding protein eIF4E play a role in translation initiation?. Eur. J. Biochem..

[32] Batool A., Aashaq S., Andrabi K.I. (2017). Reappraisal to the study of 4E-BP1 as an mTOR substrate—A normative critique. Eur. J. Cell Biol..

[33] Hay N., Sonenberg N. (2004). Upstream and downstream of mTOR. Genes Dev..

[34] Cargnello M., Tcherkezian J., Roux P.P. (2015). The expanding role of mTOR in cancer cell growth and proliferation. Mutagenesis.

[35] Jaeschke A., Hartkamp J., Saitoh M., Roworth W., Nobukuni T., Hodges A., Sampson J., Thomas G., Lamb R. (2002). Tuberous sclerosis complex tumor suppressor-mediated S6 kinase inhibition by phosphatidylinositide-3-OH kinase is mTOR independent. J. Cell Biol..

[36] Gao X., Pan D. (2001). TSC1 and TSC2 tumor suppressors antagonize insulin signaling in cell growth. Genes Dev..

[37] Inoki K., Li Y., Zhu T., Wu J., Guan K.L. (2002). TSC2 is phosphorylated and inhibited by Akt and suppresses mTOR signalling. Nat. Cell Biol..

[38] Xiao G.H., Shoarinejad F., Jin F., Golemis E.A., Yeung R.S. (1997). The tuberous sclerosis 2 gene product, tuberin, functions as a Rab5 GTPase activating protein (GAP) in modulating endocytosis. J. Biol. Chem..

[39] Saucedo L.J., Gao X., Chiarelli D.A., Li L., Pan D., Edgar B.A. (2003). Rheb promotes cell growth as a component of the insulin/TOR signalling network. Nat. Cell Biol..

[40] Guertin D.A., Sabatini D.M. (2007). Defining the role of mTOR in cancer. Cancer Cell.

[41] Pelletier J., Graff J., Ruggero D., Sonenberg N. (2015). Targeting the eIF4F translation initiation complex: A critical nexus for cancer development. Cancer Res..

[42] Kaizuka T., Hara T., Oshiro N., Kikkawa U., Yonezawa K., Takehana K., Iemura S., Natsume T., Mizushima N. (2010). Tti1 and Tel2 are critical factors in mammalian target of rapamycin complex assembly. J. Biol. Chem..

[43] Peterson T.R., Laplante M., Thoreen C.C., Sancak Y., Kang S.A., Kuehl W.M., Gray N.S., Sabatini D.M. (2009). DEPTOR is an mTOR inhibitor frequently overexpressed in multiple myeloma cells and required for their survival. Cell.

[44] Korets S.B., Czok S., Blank S.V., Curtin J.P., Schneider R.J. (2011). Targeting the mTOR/4E-BP pathway in endometrial cancer. Clin. Cancer Res..

[45] You J.S., McNally R.M., Jacobs B.L., Privett R.E., Gundermann D.M., Lin K.H., Steinert N.D., Goodman C.A., Hornberger T.A. (2019). The role of raptor in the mechanical load-induced regulation of mTOR signaling, protein synthesis, and skeletal muscle hypertrophy. FASEB J..

[46] Gruner S., Peter D., Weber R., Wohlbold L., Chung M.Y., Weichenrieder O., Valkov E., Igreja C., Izaurralde E. (2016). The Structures of eIF4E-eIF4G Complexes Reveal an Extended Interface to Regulate Translation Initiation. Mol. Cell.

[47] Mader S., Lee H., Pause A., Sonenberg N. (1995). The translation initiation factor eIF-4E binds to a common motif shared by the translation factor eIF-4 gamma and the translational repressors 4E-binding proteins. Mol. Cell. Biol..

[48] Marcotrigiano J., Gingras A.C., Sonenberg N., Burley S.K. (1999). Cap-dependent translation initiation in eukaryotes is regulated by a molecular mimic of eIF4G. Mol. Cell.

[49] Merrick W.C. (2015). eIF4F: A retrospective. J. Biol. Chem..

[50] Siddiqui N., Sonenberg N. (2015). Signalling to eIF4E in cancer. Biochem. Soc. Trans..

[51] Wang X., Li W., Williams M., Terada N., Alessi D.R., Proud C.G. (2001). Regulation of elongation factor 2 kinase by p90(RSK1) and p70 S6 kinase. EMBO J..

[52] Fu L.L., Xie T., Zhang S.Y., Liu B. (2014). Eukaryotic elongation factor-2 kinase (eEF2K): A potential therapeutic target in cancer. Apoptosis.

[53] Ryazanov A.G., Shestakova E.A., Natapov P.G. (1988). Phosphorylation of elongation factor 2 by EF-2 kinase affects rate of translation. Nature.

[54] Faller W.J., Jackson T.J., Knight J.R., Ridgway R.A., Jamieson T., Karim S.A., Jones C., Radulescu S., Huels D.J., Myant K.B. (2015). mTORC1-mediated translational elongation limits intestinal tumour initiation and growth. Nature.

[55] D’Abronzo L.S., Ghosh P.M. (2018). eIF4E Phosphorylation in Prostate Cancer. Neoplasia.

[56] Coleman L.J., Peter M.B., Teall T.J., Brannan R.A., Hanby A.M., Honarpisheh H., Shaaban A.M., Smith L., Speirs V., Verghese E.T. (2009). Combined analysis of eIF4E and 4E-binding protein expression predicts breast cancer survival and estimates eIF4E activity. Br. J. Cancer.

[57] Uniacke J., Perera J.K., Lachance G., Francisco C.B., Lee S. (2014). Cancer cells exploit eIF4E2-directed synthesis of hypoxia response proteins to drive tumor progression. Cancer Res..

[58] Hsieh A.C., Costa M., Zollo O., Davis C., Feldman M.E., Testa J.R., Meyuhas O., Shokat K.M., Ruggero D. (2010). Genetic dissection of the oncogenic mTOR pathway reveals druggable addiction to translational control via 4EBP-eIF4E. Cancer Cell.

[59] Truitt M.L., Conn C.S., Shi Z., Pang X., Tokuyasu T., Coady A.M., Seo Y., Barna M., Ruggero D. (2015). Differential Requirements for eIF4E Dose in Normal Development and Cancer. Cell.

[60] Graff J.R., Konicek B.W., Vincent T.M., Lynch R.L., Monteith D., Weir S.N., Schwier P., Capen A., Goode R.L., Dowless M.S. (2007). Therapeutic suppression of translation initiation factor eIF4E expression reduces tumor growth without toxicity. J. Clin. Invest..

[61] Galicia-Vazquez G., Cencic R., Robert F., Agenor A.Q., Pelletier J. (2012). A cellular response linking eIF4AI activity to eIF4AII transcription. RNA.

[62] Duncan R., Hershey J.W. (1983). Identification and quantitation of levels of protein synthesis initiation factors in crude HeLa cell lysates by two-dimensional polyacrylamide gel electrophoresis. J. Biol. Chem..

[63] Jones R.M., Branda J., Johnston K.A., Polymenis M., Gadd M., Rustgi A., Callanan L., Schmidt E.V. (1996). An essential E box in the promoter of the gene encoding the mRNA cap-binding protein (eukaryotic initiation factor 4E) is a target for activation by c-myc. Mol. Cell. Biol..

[64] Rosenwald I.B., Rhoads D.B., Callanan L.D., Isselbacher K.J., Schmidt E.V. (1993). Increased expression of eukaryotic translation initiation factors eIF-4E and eIF-2 alpha in response to growth induction by c-myc. Proc. Natl. Acad. Sci. USA.

[65] Lin C.J., Cencic R., Mills J.R., Robert F., Pelletier J. (2008). c-Myc and eIF4F are components of a feedforward loop that links transcription and translation. Cancer Res..

[66] Cunningham J.T., Moreno M.V., Lodi A., Ronen S.M., Ruggero D. (2014). Protein and nucleotide biosynthesis are coupled by a single rate-limiting enzyme, PRPS2, to drive cancer. Cell.

[67] Hsieh A.C., Liu Y., Edlind M.P., Ingolia N.T., Janes M.R., Sher A., Shi E.Y., Stumpf C.R., Christensen C., Bonham M.J. (2012). The translational landscape of mTOR signalling steers cancer initiation and metastasis. Nature.

[68] Joshi S., Platanias L.C. (2012). Mnk Kinases in Cytokine Signaling and Regulation of Cytokine Responses. Biomol. Concepts.

[69] Maimon A., Mogilevsky M., Shilo A., Golan-Gerstl R., Obiedat A., Ben-Hur V., Lebenthal-Loinger I., Stein I., Reich R., Beenstock J. (2014). Mnk2 alternative splicing modulates the p38-MAPK pathway and impacts Ras-induced transformation. Cell Rep..

[70] Rousseau D., Kaspar R., Rosenwald I., Gehrke L., Sonenberg N. (1996). Translation initiation of ornithine decarboxylase and nucleocytoplasmic transport of cyclin D1 mRNA are increased in cells overexpressing eukaryotic initiation factor 4E. Proc. Natl. Acad. Sci. USA.

[71] Graff J.R., Zimmer S.G. (2003). Translational control and metastatic progression: Enhanced activity of the mRNA cap-binding protein eIF-4E selectively enhances translation of metastasis-related mRNAs. Clin. Exp. Metastasis.

[72] Kevil C., Carter P., Hu B., DeBenedetti A. (1995). Translational enhancement of FGF-2 by eIF-4 factors, and alternate utilization of CUG and AUG codons for translation initiation. Oncogene.

[73] Robichaud N., del Rincon S.V., Huor B., Alain T., Petruccelli L.A., Hearnden J., Goncalves C., Grotegut S., Spruck C.H., Furic L. (2015). Phosphorylation of eIF4E promotes EMT and metastasis via translational control of SNAIL and MMP-3. Oncogene.

[74] Lazaris-Karatzas A., Montine K.S., Sonenberg N. (1990). Malignant transformation by a eukaryotic initiation factor subunit that binds to mRNA 5’ cap. Nature.

[75] Diab-Assaf M., Abou-Khouzam R., Saadallah-Zeidan N., Habib K., Bitar N., Karam W., Liagre B., Harakeh S., Azar R. (2015). Expression of eukaryotic initiation factor 4E and 4E binding protein 1 in colorectal carcinogenesis. Int. J. Clin. Exp. Pathol..

[76] Heikkinen T., Korpela T., Fagerholm R., Khan S., Aittomaki K., Heikkila P., Blomqvist C., Carpen O., Nevanlinna H. (2013). Eukaryotic translation initiation factor 4E (eIF4E) expression is associated with breast cancer tumor phenotype and predicts survival after anthracycline chemotherapy treatment. Breast Cancer Res. Treat..

[77] Zismanov V., Attar-Schneider O., Lishner M., Heffez Aizenfeld R., Tartakover Matalon S., Drucker L. (2015). Multiple myeloma proteostasis can be targeted via translation initiation factor eIF4E. Int. J. Oncol..

[78] Pettersson F., Del Rincon S.V., Emond A., Huor B., Ngan E., Ng J., Dobocan M.C., Siegel P.M., Miller W.H. (2015). Genetic and pharmacologic inhibition of eIF4E reduces breast cancer cell migration, invasion, and metastasis. Cancer Res..

[79] Zhou H., Xu R.Z., Gu Y., Shi P.F., Qian S. (2018). Targeting of phospho-eIF4E by homoharringtonine eradicates a distinct subset of human acute myeloid leukemia. Leuk Lymphoma.

[80] Volpon L., Culjkovic-Kraljacic B., Sohn H.S., Blanchet-Cohen A., Osborne M.J., Borden K.L.B. (2017). A biochemical framework for eIF4E-dependent mRNA export and nuclear recycling of the export machinery. RNA.

[81] Pettersson F., Yau C., Dobocan M.C., Culjkovic-Kraljacic B., Retrouvey H., Puckett R., Flores L.M., Krop I.E., Rousseau C., Cocolakis E. (2011). Ribavirin treatment effects on breast cancers overexpressing eIF4E, a biomarker with prognostic specificity for luminal B-type breast cancer. Clin. Cancer Res..

[82] Gao M., Zhang X., Li D., He P., Tian W., Zeng B. (2016). Expression analysis and clinical significance of eIF4E, VEGF-C, E-cadherin and MMP-2 in colorectal adenocarcinoma. Oncotarget.

[83] Salehi Z., Mashayekhi F., Shahosseini F. (2007). Significance of eIF4E expression in skin squamous cell carcinoma. Cell Biol. Int..

[84] Crew J.P., Fuggle S., Bicknell R., Cranston D.W., de Benedetti A., Harris A.L. (2000). Eukaryotic initiation factor-4E in superficial and muscle invasive bladder cancer and its correlation with vascular endothelial growth factor expression and tumour progression. Br. J. Cancer.

[85] Lu C., Makala L., Wu D., Cai Y. (2016). Targeting translation: eIF4E as an emerging anticancer drug target. Expert Rev. Mol. Med..

[86] Moerke N.J., Aktas H., Chen H., Cantel S., Reibarkh M.Y., Fahmy A., Gross J.D., Degterev A., Yuan J., Chorev M. (2007). Small-molecule inhibition of the interaction between the translation initiation factors eIF4E and eIF4G. Cell.

[87] Holm N., Byrnes K., Johnson L., Abreo F., Sehon K., Alley J., Meschonat C., Md Q.C., Li B.D. (2008). A prospective trial on initiation factor 4E (eIF4E) overexpression and cancer recurrence in node-negative breast cancer. Ann. Surg. Oncol..

[88] Kentsis A., Topisirovic I., Culjkovic B., Shao L., Borden K.L. (2004). Ribavirin suppresses eIF4E-mediated oncogenic transformation by physical mimicry of the 7-methyl guanosine mRNA cap. Proc. Natl. Acad. Sci. USA.

[89] Koromilas A.E. (2015). Roles of the translation initiation factor eIF2alpha serine 51 phosphorylation in cancer formation and treatment. Biochim. Biophys. Acta.

[90] Rajesh K., Krishnamoorthy J., Kazimierczak U., Tenkerian C., Papadakis A.I., Wang S., Huang S., Koromilas A.E. (2015). Phosphorylation of the translation initiation factor eIF2alpha at serine 51 determines the cell fate decisions of Akt in response to oxidative stress. Cell Death Dis..

[91] Wang M., Kaufman R.J. (2014). The impact of the endoplasmic reticulum protein-folding environment on cancer development. Nat. Rev. Cancer.

[92] Leprivier G., Rotblat B., Khan D., Jan E., Sorensen P.H. (2015). Stress-mediated translational control in cancer cells. Biochim. Biophys. Acta.

[93] Holcik M., Sonenberg N. (2005). Translational control in stress and apoptosis. Nat. Rev. Mol. Cell Biol..

[94] Clarke H.J., Chambers J.E., Liniker E., Marciniak S.J. (2014). Endoplasmic reticulum stress in malignancy. Cancer Cell.

[95] Proud C.G. (2001). Regulation of eukaryotic initiation factor eIF2B. Prog. Mol. Subcell Biol..

[96] Boye E., Grallert B. (2019). eIF2alpha phosphorylation and the regulation of translation. Curr Genet..

[97] Jackson R.J., Hellen C.U., Pestova T.V. (2010). The mechanism of eukaryotic translation initiation and principles of its regulation. Nat. Rev. Mol. Cell Biol..

[98] B’Chir W., Maurin A.C., Carraro V., Averous J., Jousse C., Muranishi Y., Parry L., Stepien G., Fafournoux P., Bruhat A. (2013). The eIF2alpha/ATF4 pathway is essential for stress-induced autophagy gene expression. Nucleic Acids Res..

[99] Rozpedek W., Pytel D., Mucha B., Leszczynska H., Diehl J.A., Majsterek I. (2016). The Role of the PERK/eIF2alpha/ATF4/CHOP Signaling Pathway in Tumor Progression During Endoplasmic Reticulum Stress. Curr. Mol. Med..

[100] Fernandez J., Yaman I., Sarnow P., Snider M.D., Hatzoglou M. (2002). Regulation of internal ribosomal entry site-mediated translation by phosphorylation of the translation initiation factor eIF2alpha. J. Biol. Chem..

[101] Guo L., Chi Y., Xue J., Ma L., Shao Z., Wu J. (2017). Phosphorylated eIF2alpha predicts disease-free survival in triple-negative breast cancer patients. Sci. Rep..

[102] Rosenwald I.B., Hutzler M.J., Wang S., Savas L., Fraire A.E. (2001). Expression of eukaryotic translation initiation factors 4E and 2alpha is increased frequently in bronchioloalveolar but not in squamous cell carcinomas of the lung. Cancer.

[103] Wang S., Lloyd R.V., Hutzler M.J., Rosenwald I.B., Safran M.S., Patwardhan N.A., Khan A. (2001). Expression of eukaryotic translation initiation factors 4E and 2alpha correlates with the progression of thyroid carcinoma. Thyroid.

[104] Rosenwald I.B., Koifman L., Savas L., Chen J.J., Woda B.A., Kadin M.E. (2008). Expression of the translation initiation factors eIF-4E and eIF-2* is frequently increased in neoplastic cells of Hodgkin lymphoma. Hum. Pathol..

[105] Rosenwald I.B., Wang S., Savas L., Woda B., Pullman J. (2003). Expression of translation initiation factor eIF-2alpha is increased in benign and malignant melanocytic and colonic epithelial neoplasms. Cancer.

[106] Lobo M.V., Martin M.E., Perez M.I., Alonso F.J., Redondo C., Alvarez M.I., Salinas M. (2000). Levels, phosphorylation status and cellular localization of translational factor eIF2 in gastrointestinal carcinomas. Histochem. J..

[107] Pelletier J., Thomas G., Volarevic S. (2018). Ribosome biogenesis in cancer: New players and therapeutic avenues. Nat. Rev. Cancer.

[108] Lacerda R., Menezes J., Romao L. (2017). More than just scanning: The importance of cap-independent mRNA translation initiation for cellular stress response and cancer. Cell. Mol. Life Sci..

[109] Pelletier J., Sonenberg N. (1988). Internal initiation of translation of eukaryotic mRNA directed by a sequence derived from poliovirus RNA. Nature.

[110] Hellen C.U., Sarnow P. (2001). Internal ribosome entry sites in eukaryotic mRNA molecules. Genes Dev..

[111] Shatsky I.N., Dmitriev S.E., Terenin I.M., Andreev D.E. (2010). Cap- and IRES-independent scanning mechanism of translation initiation as an alternative to the concept of cellular IRESs. Mol. Cells.

[112] Morfoisse F., Kuchnio A., Frainay C., Gomez-Brouchet A., Delisle M.B., Marzi S., Helfer A.C., Hantelys F., Pujol F., Guillermet-Guibert J. (2014). Hypoxia induces VEGF-C expression in metastatic tumor cells via a HIF-1alpha-independent translation-mediated mechanism. Cell Rep..

[113] Chen T.M., Shih Y.H., Tseng J.T., Lai M.C., Wu C.H., Li Y.H., Tsai S.J., Sun H.S. (2014). Overexpression of FGF9 in colon cancer cells is mediated by hypoxia-induced translational activation. Nucleic Acids Res..

[114] Vagner S., Gensac M.C., Maret A., Bayard F., Amalric F., Prats H., Prats A.C. (1995). Alternative translation of human fibroblast growth factor 2 mRNA occurs by internal entry of ribosomes. Mol. Cell. Biol..

[115] Martineau Y., Le Bec C., Monbrun L., Allo V., Chiu I.M., Danos O., Moine H., Prats H., Prats A.C. (2004). Internal ribosome entry site structural motifs conserved among mammalian fibroblast growth factor 1 alternatively spliced mRNAs. Mol. Cell. Biol..

[116] Stein I., Itin A., Einat P., Skaliter R., Grossman Z., Keshet E. (1998). Translation of vascular endothelial growth factor mRNA by internal ribosome entry: Implications for translation under hypoxia. Mol. Cell. Biol..

[117] Weingarten-Gabbay S., Elias-Kirma S., Nir R., Gritsenko A.A., Stern-Ginossar N., Yakhini Z., Weinberger A., Segal E. (2016). Comparative genetics. Systematic discovery of cap-independent translation sequences in human and viral genomes. Science.

[118] Hershey J.W., Sonenberg N., Mathews M.B. (2012). Principles of translational control: An overview. Cold Spring Harb. Perspect. Biol..

[119] Sriram A., Bohlen J., Teleman A.A. (2018). Translation acrobatics: How cancer cells exploit alternate modes of translational initiation. EMBO Rep..

[120] Shi Y., Yang Y., Hoang B., Bardeleben C., Holmes B., Gera J., Lichtenstein A. (2016). Therapeutic potential of targeting IRES-dependent c-myc translation in multiple myeloma cells during ER stress. Oncogene.

[121] Bert A.G., Grepin R., Vadas M.A., Goodall G.J. (2006). Assessing IRES activity in the HIF-1alpha and other cellular 5′ UTRs. RNA.

[122] Fernandez J., Bode B., Koromilas A., Diehl J.A., Krukovets I., Snider M.D., Hatzoglou M. (2002). Translation mediated by the internal ribosome entry site of the cat-1 mRNA is regulated by glucose availability in a PERK kinase-dependent manner. J. Biol. Chem..

[123] Gonzalez-Herrera I.G., Prado-Lourenco L., Teshima-Kondo S., Kondo K., Cabon F., Arnal J.F., Bayard F., Prats A.C. (2006). IRES-dependent regulation of FGF-2 mRNA translation in pathophysiological conditions in the mouse. Biochem. Soc. Trans..

[124] Shatsky I.N., Terenin I.M., Smirnova V.V., Andreev D.E. (2018). Cap-Independent Translation: What’s in a Name?. Trends Biochem. Sci..

[125] Blanco-Perez M., Perez-Canamas M., Ruiz L., Hernandez C. (2016). Efficient Translation of Pelargonium line pattern virus RNAs Relies on a TED-Like 3 -Translational Enhancer that Communicates with the Corresponding 5 -Region through a Long-Distance RNA-RNA Interaction. PLoS ONE.

[126] Andreev D.E., Dmitriev S.E., Zinovkin R., Terenin I.M., Shatsky I.N. (2012). The 5′ untranslated region of Apaf-1 mRNA directs translation under apoptosis conditions via a 5’ end-dependent scanning mechanism. FEBS Lett..

[127] Signer R.A., Magee J.A., Salic A., Morrison S.J. (2014). Haematopoietic stem cells require a highly regulated protein synthesis rate. Nature.

[128] Signer R.A., Qi L., Zhao Z., Thompson D., Sigova A.A., Fan Z.P., DeMartino G.N., Young R.A., Sonenberg N., Morrison S.J. (2016). The rate of protein synthesis in hematopoietic stem cells is limited partly by 4E-BPs. Genes Dev..

[129] Kim H.J., McMillan E., Han F., Svendsen C.N. (2009). Regionally specified human neural progenitor cells derived from the mesencephalon and forebrain undergo increased neurogenesis following overexpression of ASCL1. Stem Cells.

[130] Wright L.S., Li J., Caldwell M.A., Wallace K., Johnson J.A., Svendsen C.N. (2003). Gene expression in human neural stem cells: Effects of leukemia inhibitory factor. J. Neurochem..

[131] Vogel C., Marcotte E.M. (2012). Insights into the regulation of protein abundance from proteomic and transcriptomic analyses. Nat. Rev. Genet..

[132] Tahmasebi S., Jafarnejad S.M., Tam I.S., Gonatopoulos-Pournatzis T., Matta-Camacho E., Tsukumo Y., Yanagiya A., Li W., Atlasi Y., Caron M. (2016). Control of embryonic stem cell self-renewal and differentiation via coordinated alternative splicing and translation of YY2. Proc. Natl. Acad. Sci. USA.

[133] Ingolia N.T., Lareau L.F., Weissman J.S. (2011). Ribosome profiling of mouse embryonic stem cells reveals the complexity and dynamics of mammalian proteomes. Cell.

[134] Barna M., Ruggero D. (2014). Tailor made protein synthesis for HSCs. Cell Stem Cell.

[135] Blanco S., Bandiera R., Popis M., Hussain S., Lombard P., Aleksic J., Sajini A., Tanna H., Cortes-Garrido R., Gkatza N. (2016). Stem cell function and stress response are controlled by protein synthesis. Nature.

[136] Sanchez C.G., Teixeira F.K., Czech B., Preall J.B., Zamparini A.L., Seifert J.R., Malone C.D., Hannon G.J., Lehmann R. (2016). Regulation of Ribosome Biogenesis and Protein Synthesis Controls Germline Stem Cell Differentiation. Cell Stem Cell.

[137] Browning K.S., Maia D.M., Lax S.R., Ravel J.M. (1987). Identification of a new protein synthesis initiation factor from wheat germ. J. Biol. Chem..

[138] Rhoads R.E. (2009). eIF4E: New family members, new binding partners, new roles. J. Biol. Chem..

[139] Uniacke J., Holterman C.E., Lachance G., Franovic A., Jacob M.D., Fabian M.R., Payette J., Holcik M., Pause A., Lee S. (2012). An oxygen-regulated switch in the protein synthesis machinery. Nature.

[140] Osborne M.J., Volpon L., Kornblatt J.A., Culjkovic-Kraljacic B., Baguet A., Borden K.L. (2013). eIF4E3 acts as a tumor suppressor by utilizing an atypical mode of methyl-7-guanosine cap recognition. Proc. Natl. Acad. Sci. USA.

[141] Yang G., Smibert C.A., Kaplan D.R., Miller F.D. (2014). An eIF4E1/4E-T complex determines the genesis of neurons from precursors by translationally repressing a proneurogenic transcription program. Neuron.

[142] Yoffe Y., David M., Kalaora R., Povodovski L., Friedlander G., Feldmesser E., Ainbinder E., Saada A., Bialik S., Kimchi A. (2016). Cap-independent translation by DAP5 controls cell fate decisions in human embryonic stem cells. Genes Dev..

[143] Imataka H., Gradi A., Sonenberg N. (1998). A newly identified N-terminal amino acid sequence of human eIF4G binds poly(A)-binding protein and functions in poly(A)-dependent translation. EMBO J..

[144] Lamphear B.J., Kirchweger R., Skern T., Rhoads R.E. (1995). Mapping of functional domains in eukaryotic protein synthesis initiation factor 4G (eIF4G) with picornaviral proteases. Implications for cap-dependent and cap-independent translational initiation. J. Biol. Chem..

[145] Sugiyama H., Takahashi K., Yamamoto T., Iwasaki M., Narita M., Nakamura M., Rand T.A., Nakagawa M., Watanabe A., Yamanaka S. (2017). Nat1 promotes translation of specific proteins that induce differentiation of mouse embryonic stem cells. Proc. Natl. Acad. Sci. USA.

[146] Yamanaka S., Zhang X.Y., Maeda M., Miura K., Wang S., Farese R.V., Iwao H., Innerarity T.L. (2000). Essential role of NAT1/p97/DAP5 in embryonic differentiation and the retinoic acid pathway. EMBO J..

